# Monitoring of Surveillance Quality Indicators of Measles in Iranian Districts: Analysis of Measles Surveillance System 2014-2016

**Published:** 2018-06-30

**Authors:** Seyed Mohsen Zahraei, Abolfazl Mohammadbeigi, Narges Mohammadsalehi, Azam Sabouri, Sima Afrashteh, Shahram Arsang Jang, Hossein Ansari, Salman Khazaei

**Affiliations:** ^1^ Center for Communicable Diseases Control, Ministry of Health and Medical Education, Tehran, Iran; ^2^ Neurology and Neurosciences Research Center, Qom University of Medical Sciences, Qom, Iran; ^3^ Vic-Chancellor for Public Health, Bushehr University of Medical Sciences, Bushehr, Iran; ^4^ Research Center for Environmental Pollutants, Qom University of Medical Sciences, Qom, Iran; ^5^ Health Promotion Research Center, Department of Epidemiology and Biostatistics, Zahedan University of Medical Sciences, Zahedan, Iran; ^6^ Department of Epidemiology, School of Public Health, Hamadan University of Medical Sciences, Hamadan, Iran

**Keywords:** Elimination, Iran, Measles, Vaccination, Surveillance

## Abstract

**Background:** The elimination target for measles as an acute and contagious disease in Eastern Mediterranean Region (EMR) and Iran is planned by high-quality surveillance. We aimed to monitor the surveillance quality indicators of measles by in all districts of Iran during 2014-16.

**Study design:** A cross-sectional study.

**Methods:** Four quality surveillance indicators of measles including non measles discarded rate, percent of suspected cases with adequate investigation, percent of adequate blood specimen collection and percent with timely availability of laboratory results were assessed in Iran. Surveillance data of measles were extracted from the measles surveillance system and the risk point score for each district was calculated based on WHO Risk Assessment Tool by a function of four indicators.

**Results:** Overall, 14312 suspected cases and 322 districts were assessed and the risk points of measles' quality surveillance showed that 92.8% of Iranian districts were categorized as low risk, 2.8% medium risk, 0.62% high risk and 3.73% very high-risk category. The appropriate non measles discarded rate indicator was 87.3%. The percent of suspected cases with adequate investigation (more than 2 per 100000 people) was 87.9%. Moreover, the average of percent adequate blood specimen collection and percent with timely availability of laboratory results was 85.16% and 85.71%, respectively in all Iranian districts.

**Conclusions:** The surveillance quality indicators in Iran were good and higher than the WHO plans. Increasing the percentage of non-measles discarded rate could improve the poor quality in high risk and very high-risk districts.

## Introduction

Measles is an acute and highly contagious respiratory disease^1,2^ that is the cause of serious illness, lifelong complications and more than two million deaths annually worldwide ^3,4^. During 2000 to 2010, the estimated global measles mortality of measles decreased to 74% from 535300 deaths to 139300 ^3^. Measles is still left over as an important public health problem in Iran that is a member of the Eastern Mediterranean Region (EMR) of WHO.^1^ However, the elimination target for measles in EMR region planned for 2010 as the interruption of endemic measles virus transmission for more than one year by high quality surveillance ^1,3^.

The programs for elimination of measles in Iran are including the two-stage of children immunization 12 and 18 months after birth, mass immunization of 5 to 25 yr old against measles/rubella at 2003, Supplementary Immunization Activities (SIAs) in deprived and high risk areas and enhanced high-quality case-based measles surveillance by timely and accurate testing of specimens^5-8^. Based on national elimination practices in Iran, the overall coverage of full vaccines immunization is 96.8%^9^ and the vaccination coverage for first and second dose of MMR in under 5-yr-old children in suburb area of big Iranian cities was estimated as 97.1% and 94.9% respectively ^9,10^ and SIAs in Iranian target population children was estimated as 98.7%^11^. However, despite these achievements, progress toward elimination goals has been slowed and measles occurrence happened in some areas.

In 2012, WHO Regional Committees reaffirmed its commitment to eliminate measles to interrupt endemic measles virus transmission as rapidly as possible^12^. To prevent future measles outbreaks a new tool was developed to assess district-level risk for measles outbreaks. The WHO measles programmatic risk assessment tool estimate the risk point of measles for each districts^13-15^. “Measles elimination programs need to be monitored through the analysis of surveillance data and performance indicators”^16^. Therefore, we aimed to monitor the surveillance quality indicators of measles by estimating and mapping the risk of measles outbreak in all districts of Iran based measles programmatic risk assessment tool during 2014-16.

## Methods

In this cross-sectional study based on retrospective existing data, the indicators of measles surveillance during 2014-2016 were assessed in Iran and all districts of Iran were considered as the study subjects.

### Measles surveillance in Iran

The measles surveillance in Iran is a robust communicable disease surveillance system supported by laws relating to mandatory reporting of all suspected measles cases immediately. The surveillance system of measles is responsible for collecting and reporting surveillance data and managed by a network of medical universities in Iran. WHO standard recommendations for measles surveillance used as national guidelines for measles surveillance in elimination phase and distributed to all health facilities to detect and report all cases^7^.

### Data Sources

We used from 4 different data sources including areas of each Iranian district in Geographic Information System (GIS) shapefile, population census of 2016 for each district, vaccination coverage for measles and Pentavalent during study years. Moreover, surveillance data of measles were extracted from the measles surveillance system in Center for Communicable Diseases Control (CCDC) in Ministry of Health. All the suspected cases of measles with clinical findings including fever, generalized maculopapular rash and either a cough, coryza or conjunctivitis^17^ that categorized as Lab-Confirmed Measles,‏ Epi-Linked Measles,‏ clinically Compatible Measles, Confirmed Rubella, Discarded‏ and‏ Pending during 2014-2016. Moreover, 14 variables regarding to measles monitoring including year, province, reporting district, case id, final classification, age in years, age in months, sex, place of residence, date of rash onset, vaccination status, number of vaccine doses, date of notification, date of investigation, date of blood sample collection, date district received lab result and place of infection or travel history‏ were collected for each suspected cases in surveillance system. These variables used to calculate the surveillance quality indicators based WHO Risk Assessment Tool^18^.

Surveillance quality was assessed as a function of combined indicator scores from four indicators. Surveillance quality evaluates the ability of a district to detect and confirm cases rapidly and accurately. These indicators including four items: 1) the non-measles discarded rate, as equal to the number of non-measles discarded cases divided by the population per 100000 for each year; 2) the proportion of suspected measles cases with adequate investigation (investigation within 48 h of notification and inclusion of 10 core variables); 3) the proportion of cases with adequate specimen collection (within 28 d of rash onset); and 4) the proportion of cases for whom laboratory results were available in a timely manner ^19^. The maximum risk points for non-measles discarded rate indicator is 8 and other three indicators could take the maximum risk 4. Therefore, the overall possible risk points for each district in surveillance quality was 20 and it was calculated by the WHO measles programmatic risk assessment tool. Then the overall risk of surveillance quality of measles calculated and the tool assigned each district a risk category of low, medium, high, or very high and mapped it^12,15^. To establish cut-off criteria for risk categories, a distribution was constructed consisting of all possible combinations of scores from each indicator. Then, the risk scoring categories are defined by the 50th, 75th, and 90th percentiles of this distribution^19^. The program delivery performance indicator for each district was estimated by drop-out rate MCV1-MCV2 and DPT1-MCV1.

### Data Analysis

Data were analyzed in WHO Measles Programmatic Risk Assessment Tool that works under Excel and GIS software (ver. 9.3). We used a later version of shapefile for Iran that contains 322 districts because the new updated shapefile for all districts of Iran did not found. Therefore, some new districts (cities) separated recently, are merged with the prior districts. The risk point score for surveillance quality in each Iranian district was calculated based on WHO Risk Assessment Tool^18,20^.

## Results

Overall, 14312 suspected cases were assessed and 22.1% (3165), 45.9% (6573) and 32% (4574) were registered from 2014 to 2016, respectively. Overall, 322 districts in 31 provinces of Iran were assessed and the risk points of measles outbreak were calculated based on four different surveillance quality indicators. The appropriate non measles discarded rate indicators were 87.3%, the percent of suspected cases with adequate investigation (more than 2 per 100000 people) was 87.9%. Moreover, the average of percent adequate blood specimen collection and percent with timely availability of laboratory results was 85.16% and 85.71%, respectively in all Iranian districts.

**Figure 1:**
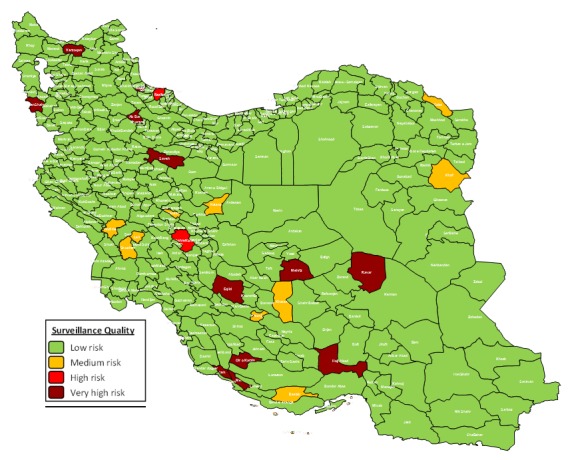
The Mapping of the Surveillance Quality risk points in Iranian districts 2017

The surveillance quality in most districts (92.86%) of Iran is categorized as low risk. However, 9 districts (2.8%) were in medium risk category. Two districts (0.62%) categorized as high risk and 12 districts (3.73%) as very high-risk category. Based on Figure 1 very high-risk districts included Jam in Bushehr, Varzaqan in Esat Azerbaijan, Eqlid, Mohr and Qir o Karzin in Fars, Masal in Gilan, Haji Abad in Hormozgan, Ravar in Kerman, Saveh in Markazi, PiranShahr in West Azerbaijan, Mehriz in Yazd and Soltanieh in Zanjan provinces. In addition, Shahrkord and Lahijan districts were categorized as high-risk category. Khaf, Kalat, Andimeshk, Lali, Shushtar, Khansar, Natanz, Arsanjan, Khatam, and Bastak were categorized as medium risk category.

**Figure 2:**
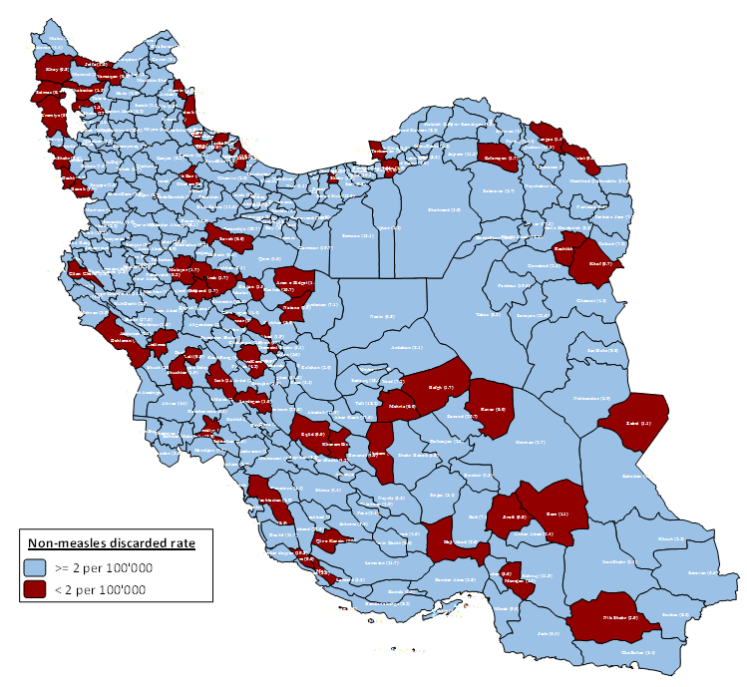
Non-measles discarded rate indicator in Iranian districts

The non measles discarded rate in all districts were more than 2 per 100000 people except in 43 districts (12.7%) as including, Namin, Jam, Lordegan, ShahreKord, Jolfa, Osku, Shabestar, Varzaqan, Eqlid, Mohr, Qir and Karzin, Langrud, Masal, Rasht, RudSar, Talesh, Bandar Torkaman, Gorgan, Malayer, Aran and Bidgol, Najaf Abad, Bam, Ravar, Esfarayen, Andimeshk, Izeh, Omidiye, Shushtar, Baneh, Arak, Saveh, Shazand, Ghaem Shahr, Nik Shahr, Zabol, PiranShahr, Salmas, SarDasht, Urumia, Mehriz and Soltanieh (Figure 2). The details of risk points and districts with non-measles discarded rate lower than 2 per 100000 people is presented in Table 1.

**Table 1:** The Surveillance indicators for measles in districts with risk point (RP) higher than 4 in Iran, 2017

**Area**	**% non-measles** **discarded rate**		**% with adequate** **investigation**		**% adequate blood** **specimen collection**		**% with timely availability** ** of laboratory results**		**Total RP**
	**2016**	**RP**	**2016**	**RP**	**2016**	**RP**	**2016**	**RP**	
Bushehr									
Dailam	5.7	0	50	4	50	4	100	0	8
Dashtestan	2.0	4	100	0	100	0	100	0	4
Jam	0.0	8	0	4	0	4	0	4	20
Charmahal									
Lordegan	1.0	8	100	0	100	0	100	0	8
ShahreKord	0.9	8	67	4	67	4	100	0	16
Esat.Azerbaijan									
Osku	1.3	4	100	0	100	0	100	0	4
Shabestar	1.5	4	50	4	100	0	100	0	8
Varzaqan	0.0	8	0	4	0	4	0	4	20
Fars									
Arsanjan	9.4	0	75	4	75	4	67	4	12
Darab	4.0	0	100	0	100	0	25	4	4
Eqlid	0.0	8	0	4	0	4	0	4	20
Estahban	2.9	0	100	0	100	0	50	4	4
FirozAbad	3.4	0	25	4	25	4	100	0	8
Mamasani	4.4	0	86	0	100	0	71	4	4
Mohr	0.0	8	0	4	0	4	0	4	20
Qir o Karzin	0.0	8	0	4	0	4	0	4	20
Shiraz	4.4	0	78	4	78	4	92	0	8
Gilan									
Fuman	1.1	0	0	4	100	0	100	0	4
Lahijan	2.4	0	75	4	100	0	100	0	4
Langrud	0.7	8	100	0	100	0	100	0	8
Masal	0.0	8	0	4	0	4	0	4	20
Rasht	0.2	8	33	4	33	4	100	0	16
RudSar	1.3	4	100	0	100	0	100	0	4
Talesh	1.0	8	100	0	100	0	100	0	8
Golestan									
Bandar Torkaman	0.7	8	100	0	100	0	100	0	8
Gorgan	1.9	4	100	0	100	0	100	0	4
Hamedan									
Hamadan	9.2	0	100	0	100	0	73	4	4
Malayer	1.7	4	100	0	100	0	100	0	4
Hormozgan									
Aboo Mosa	0.0	0	0	4	0	4	0	4	12
Bandar Abas	3.8	0	56	4	100	0	83	0	4
Bandar Lengeh	8.2	0	100	0	100	0	58	4	4
Bastak	2.5	0	50	4	50	4	0	4	12
Haji Abad	0.0	8	0	4	0	4	0	4	20
Hengam	0.0	0	0	4	0	4	0	4	12
Jask	6.4	0	100	0	100	0	71	4	4
Kish	0.0	0	0	4	0	4	0	4	12
Qeshm	4.0	0	100	0	100	0	50	4	4
Ilam									
Shirvan Chardoval	11.1	0	88	0	100	0	75	4	4
Isfahan									
Aran o Bidgol	1.9	4	100	0	100	0	100	0	4
Khansar	0.0	0	0	4	0	4	0	4	12
Najaf Abad	1.9	4	100	0	100	0	100	0	4
Natanz	0.0	0	0	4	0	4	0	4	12
Kerman									
Bam	1.1	4	100	0	100	0	71	4	8
Jiroft	0.8	8	100	0	100	0	100	0	8
Ravar	0.0	8	0	4	0	4	0	4	20
Khorasan.Jonobi									
SarBishe	9.8	0	75	4	100	0	100	0	4
Khorasan.Razavi									
Fariman	9.1	0	89	0	89	0	75	4	4
Kalat	0.0	0	0	4	0	4	0	4	12
Khaf	0.7	8	100	0	100	0	0	4	12
Khalil Abad	15.5	0	75	4	75	4	100	0	8
Sabzevar	2.7	0	58	4	100	0	92	0	4
Khorasan.Shomali									
Esfarayen	1.7	4	50	4	100	0	100	0	8
Farouj	26.4	0	100	0	100	0	77	4	4
Khuzestan									
Abadan	3.0	0	67	4	67	4	83	0	8
Andimeshk	1.2	4	50	4	50	4	100	0	12
Izeh	1.0	4	100	0	100	0	100	0	4
KhoramShahr	5.8	0	80	0	70	4	63	4	8
Lali	0.0	0	0	4	0	4	0	4	12
Omidiye	1.8	4	80	0	133	0	100	0	4
Shushtar	1.9	4	60	4	40	4	100	0	12
Kordestan									
Baneh	1.9	4	100	0	100	0	100	0	4
Lorestan									
PolDokhtar	5.5	0	75	4	75	4	100	0	8
Markazi									
Arak	1.7	4	100	0	100	0	100	0	4
Saveh	0.0	8	0	4	0	4	0	4	20
Shazand	1.7	4	100	0	100	0	100	0	4
Chalus	2.1	0	100	0	100	0	75	4	4
Ghaem Shahr	1.6	4	100	0	100	0	100	0	4
Juybar	3.9	0	67	4	67	4	100	0	8
Mahmud Abad	2.3	0	100	0	100	0	67	4	4
Nur	4.1	0	60	4	100	0	100	0	4
Shahrood	3.9	0	100	0	100	0	100	0	4
Sistan-O-Bluchestan									
Nik Shahr	2.0	4	100	0	100	0	100	0	4
Zabol	1.1	4	100	0	100	0	100	0	4
Khoy	0.8	8	100	0	100	0	100	0	8
PiranShahr	0.0	8	0	4	0	4	0	4	20
Salmas	0.5	8	100	0	100	0	100	0	8
SarDasht	0.8	8	100	0	100	0	100	0	8
ShahinDej	5.4	0	71	4	71	4	100	0	8
Uromiye	0.9	8	100	0	100	0	100	0	8
Yazd									
Khatam	0.0	0	0	4	0	4	0	4	12
Mehriz	0.0	8	0	4	0	4	0	4	20
Zanjan									
Soltanieh	0.0	8	0	4	0	4	0	4	20

**Figure 3:**
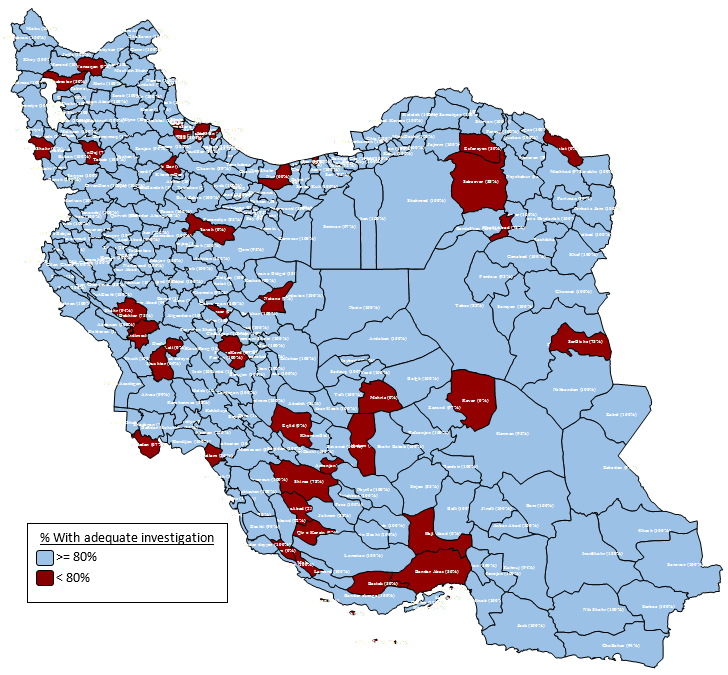
Percent of suspected cases with adequate investigation in Iranian districts

The percent of suspected cases with adequate investigation (Figure 3) was higher as 80% in all districts of Iran except in 39 districts (12.11%) including Dailam, Jam, Shahrekord, Varzaqan, Arsanjan, Eqlid, FirozAbad, Mohr, Qir and Karzinm, Shiraz, Fuman, Lahijan, Masal, Rasht, Aboo Mosa, Bandar Abas, Bastak, Haji Abad, Hengam, Kish, Natanz, Ravar, SarBishe, Kalat, Khalil Abad, Esfarayen, Sabzevar, Abadan, Andimeshk, Lali, Shushtar, PolDokhtar, Saveh, Juybar, Nur, PiranShahr, ShahinDej, Khatam, Mehriz and Soltanieh.

Table 1, shows the risk point of measles in all districts with at least one weak indicators in surveillance of measles. Some districts have minimum risk point equal zero that did not present in Table 1. However, more than of Iranian districts have the best surveillance quality and did not bring in table. Districts with higher risk points need to more monitoring and should promote the indicators for measles surveillance.

In addition, the program delivery performance indicator showed that all districts in Iran were categorized as low risk area. We have not any district in Iran that received full risk points for program delivery performance indicator. The drop-out rate MCV1-MCV2 and DPT1-MCV1 was lower than 10% in all districts of Iran between 2014-2016.

## Discussion

The surveillance quality in most districts of Iran was good based on the WHO measles programmatic risk assessment tool and 93% of Iranian districts were in low risk category and only 4.35% of districts were categorized as high risk and very high risk. This method of risk assessment for measles used in other studies in India ^21^, Namibia ^13^, Romania ^14^, and Philippines ^12^. In comparison to these studies the status of Iran in control and elimination of measles in very good and the risk point score of surveillance quality in Iran is acceptable.

The high and very high-risk districts were cities that located in a belt from North West to South East. The Fars Province with three very high-risk districts including Eqlid, Mohr and Qir and Karzin showed the lower surveillance quality. Moreover, 9 (2.79%) Iranian districts received full risk point of surveillance quality. The highest risk point for surveillance quality was equal 20 and theses districts reached 20. However, the poor quality in high risk and very high-risk categories are related to low percentage of non-measles discarded rate as the most important factor in Iran.

Our study showed that the appropriate non measles discarded rate indicators, the percent of suspected cases with adequate investigation, the average of percent adequate blood specimen collection and percent with timely availability of laboratory results were lower 88% in Iran. In China, the percentage of suspected measles cases investigated within 48 h of reporting was 93.30% and the percentage of suspected measles cases providing an adequate serum specimen estimated 90.37%. Moreover, the percentage of serum specimens with laboratory results reported within 7 d of specimen collection was 93.85% at 2011^4,16^. The surveillance quality in Iran was lower than China.

Moreover, the based program delivery performance indicator, all districts in Iran were in low risk area. This indicator affected by MCV1-MCV2 and DPT1 vaccination coverage and the drop-out rate MCV1-MCV2 and DPT1-MCV1. However, other recent studies in Iran showed a high immunization coverage for all routine vaccines in Iran especially for MCV1 and MCV2 and DPT1/Pantavalan^9,22^.

However, the surveillance quality in Iran was very good except in some cities. Therefore, the health providers in each district should decrease the percentage of suspected measles cases that are unvaccinated or unknown vaccination status. Based on WHO plans, the quality surveillance indicators including adequacy of investigation, reporting rate of discarded non-measles, laboratory confirmation and timeliness of case reporting should be higher 80% ^23,24^ and our indicators were higher. Nevertheless, the surveillance quality should be improved in Iran by the following items, especially by the following priority. First, the non-measles discarded rate indicators should be improved and increased to higher 95% in Iran. Second, the proportion of suspected measles cases with adequate investigation by 10 core variables during 48 h of notification should be improved and increased to higher 95% in Iran. Third, increase the ability of a district to detect and confirm cases with available laboratory results at a defined time, rapidly and accurately after onset of disease. Forth, the proportion of cases with adequate specimen collection within 28 d of rash onset should be increased^3,23,25^. Moreover, SIAs as one of strategic key towards elimination of measles could restore the lacks in immunization coverage in districts with high immigration form neighborhood countries as it is showed in other studies ^8,11,23^.

Todays, 74% reduction in measles-related mortality is registered worldwide between 2000 and 2010^26^ and measles in Iran is reached to elimination phase from 2012 ^1^ and the measles immunization coverage has increased from 38% in 1980 to 99% ^1,5,9^. However, new cases of measles in Iran with source-imported cases from neighbor countries with endemic activity of measles could distort the elimination activities of Iran.

However, our study had some limitations. The updated shapefile of all Iranian districts could be more effective for better conclusion about districts with weak surveillance quality. Nevertheless, collective efforts of Iran with neighborhood countries including Iraq, Pakistan, and Afghanistan are important for achieving to measles elimination^8^. However, the WHO measles risk assessment tool did not consider the magnitude of reproductive rate and size of measles outbreaks in the overall risk and surveillance quality while this index is a proxy for quality of surveillance system. The activities regarding strengthen immunization programs to achieve high population immunity maintain high-quality surveillance for rapid case detection and confirmation should be address regional.

## Conclusion

The surveillance quality in 93% of Iranian districts was good. The Fars Province with three very high-risk districts has the lower surveillance quality. Moreover, only 2.79% of Iranian districts received full risk point of surveillance quality. However, increasing the percentage of non-measles discarded rate as the most important factor in Iran could improve the poor quality in high risk and very high-risk districts. Besides keeping high immunization coverage of MCV1, MCV2, and DPT1/Pentavalent, the CCDC of each district should enhance the percent of suspected cases with adequate investigation, the average of percent adequate blood specimen collection and percent with timely availability of laboratory results.

## Acknowledgements

The authors are very grateful for WHO Office in Iran Collaboration Center and Center for Communicable Diseases Control in the Ministry of Health and Medical Education for preparing data.

## Conflict of interest statement

The authors declare that there is no conflict of interest.

## Funding

WHO Office in Iran Collaboration Center.

## Highlights

- The risk points of measles' quality surveillance showed that 92.86% of Iranian districts are categorized as low risk.
- Four indicators of quality surveillance for measles in Iran were higher than 80%, upper than the WHO plans.
- The program delivery performance indicator showed that all districts in Iran were categorized as low risk area.
- The drop-out rate MCV1-MCV2 and DPT1-MCV1 was lower than 10% in all districts of Iran, which is acceptable.
